# Infant-Type Bifidobacteria as Biocontrol Agents: Suppressing *Listeria monocytogenes* via Key Virulence Gene Alteration

**DOI:** 10.1155/cjid/6638044

**Published:** 2025-12-03

**Authors:** Qianglai Tan, Yiting Zheng, Lanlan Wu, Yanling Wang, Wanqian Xu, Muding Lin, Zhen Zeng

**Affiliations:** ^1^Department of Public Health and Medical Technology, Xiamen Medical College, Xiamen 361023, Fujian, China; ^2^Department of Pharmacy, Xiamen Medical College, Xiamen 361023, Fujian, China

**Keywords:** antibacterial, bifidobacteria, *Listeria monocytogenes*, RNA-seq, virulence gene expression

## Abstract

Infant-type bifidobacteria exhibit potential as targeted biocontrol agents against *Listeria monocytogenes* infections. However, the strain-specific antimicrobial efficacy and molecular mechanisms remain insufficiently characterized. In this study, the probiotic potential of five bifidobacteria strains was systematically evaluated through comprehensive *in vitro* antioxidant and antibacterial activities. Furthermore, RNA-seq analysis was employed to elucidate the global transcriptomic responses of *L. monocytogenes* during coculture with three selected bifidobacteria strains. The results revealed that the tested bifidobacteria strains possess significant *in vitro* antioxidant and antibacterial activities. Notably, three infant-type bifidobacteria strains exhibited particularly potent growth inhibition against *L. monocytogenes*. Co-incubation with these strains significantly reduced the survival of *L. monocytogenes* and modulated the expression of key genes. A substantial proportion of differentially expressed genes (DEGs) were enriched in pathways related to anion binding, ABC transporters, quorum sensing, peptidoglycan biosynthesis, and glutathione metabolism, suggesting common mechanisms underlying the inhibitory effects of infant-type bifidobacteria strains. Furthermore, several virulence-associated DEGs, including *ahpC*, *clpC*, *clpX*, *recN*, *arlR*, *rpoD*, *tlyA*, and *hlyIII*, were identified as shared antagonistic targets. Strain-specific DEGs, such as *msrB*, *amiE*, *clpE*, and *clpL*, were also identified, reflecting variations in virulence inhibition and stress-response mechanisms. These findings advance our understanding of probiotics–pathogen interactions and provide a foundation for developing targeted strategies to combat *L. monocytogenes*.

## 1. Introduction

*Listeria monocytogenes* is a well-documented foodborne pathogen capable of causing severe invasive infections, particularly in immunocompromised individuals [1]. It can lead to serious clinical outcomes, including listeriosis, gastroenteritis, meningitis, and abortion [2]. The high mortality rate among vulnerable populations poses a significant public health challenge. Surveillance data reveal 211 listeriosis cases reported from 64 sentinel hospitals in China (2013–2017) [3]. In the United States, an estimated 1600 infections annually result in approximately 260 deaths, primarily affecting the elderly, pregnant women, and newborns [4]. *L. monocytogenes* exhibits exceptional adaptability, enabling its survival and transmission through food, environmental sources, and the host gastrointestinal tract. This resilience is primarily mediated by transcriptional regulation of stress response and virulence mechanisms. For instance, Wu et al. [5] characterized GadR, a novel RofA-family transcriptional regulator, as a key determinant of acid resistance. Additionally, GshF promotes oxidative stress tolerance by altering carbohydrate and amino acid metabolism [6]. The two-component system LiaSR has also been shown to negatively modulate acid resistance and virulence in *L. monocytogenes* [7]. Additionally, Phelps et al. [8] highlighted the essential role of virulence factors, including Listeriolysin O, InlA, and InlB, in breaching host barriers and facilitating tissue invasion. Collectively, these insights highlight the pressing need for targeted interventions to control *L. monocytogenes* infections and reduce its pathogenic potential.

In recent decades, the assessment and utilization of bioprotective agents against pathogenic microorganisms have been a major focus of intensive research. These agents exert their protective effects primarily through antimicrobial compound production and modulation of critical gene expression pathways. Lactic acid bacteria are widely recognized as effective natural biopreservatives for controlling the growth of *L. monocytogenes* in food products [9]. For instance, *Enterococcus faecium* B1 has been shown to reduce the survival of *L. monocytogenes* and inhibit the expression of virulence genes such as *prfA*, *hly*, *inlA*, and *inlB* virulence genes [10]. Similarly, Rios-Covian et al. [11] reported that *Bifidobacterium breve* IPLA20005 modulates the expression of *hly* and *luxS* virulence genes in *L. monocytogenes*. Bifidobacteria strains are considered key traditional probiotics and are commonly used to prevent and treat enteric infections [12]. The genus exhibits broad-spectrum antimicrobial activity against diverse pathogens in both *in vitro* and *in vivo* systems [13]. Notably, *B. adolescentis* exhibits strong antagonistic activity against *Candida albicans* [14]. Roozbahani et al. [15] demonstrated that *B. animalis* subsp. *lactis* BB-12 and its cell-free supernatant (CFS) effectively inhibit the growth of *L. monocytogenes* and *Salmonella enterica* serovar Typhimurium. Our prior work revealed that *B. longum* NCC2705 alters transcriptional regulation of multiple *L. monocytogenes* virulence genes during coculture [16]. However, systematic comparisons of *L. monocytogenes* global transcriptomic responses to different bifidobacteria strains remain scarce. Therefore, this study aimed to investigate the probiotic potential of five bifidobacteria strains through *in vitro* antioxidant and antibacterial activity assays. Furthermore, RNA-seq transcriptomics was employed to characterize differential gene expression profiles in *L. monocytogenes* during coculture with selected bifidobacteria strains. This integrated approach provides mechanistic insights into probiotic–pathogen interactions, elucidates strain-specific antagonistic mechanisms, and informs the development of targeted antimicrobial strategies.

## 2. Methods

### 2.1. Bacterial Strains and Growth Conditions

*L. monocytogenes* National Center for Medical Culture Collections (CMCC) 54002 and five bifidobacteria strains, including *B. breve* SHBCC D11205, *B. bifidum* GDMCC 1.324, *B. longum* subsp. *infantis* GDMCC 1.1258, *B. animalis* subsp. *animalis* GDMCC 1.169, and *B. animalis* subsp. *Lactis* SHBCC D15854 used in this study, were purchased from CMCC, the Shanghai Bioresource Collection Center (SHBCC), and the Guangdong Microbial Culture Collection Center (GDMCC), respectively. Both bacterial stocks were stored at −80°C in appropriate media supplemented with 30% (v/v) glycerol and subcultured twice on fresh agar plates before use. A single colony of *L. monocytogenes* CMCC 54002 was aerobically subcultured in Trypticase soy broth (TSB) (Huankai, Guangdong, China) with shaking at 180 rpm and incubated at 37°C for 24 h. The five bifidobacteria strains were cultured anaerobically in de Man, Rogosa, and Sharpe Broth (MRS) (Solarbio, Beijing, China) at 37°C for 48 h.

### 2.2. Preparation of CFS

After incubation under the previously mentioned conditions, the bifidobacteria CFS was collected by centrifugation at 8000 rpm for 15 min and filtered through a syringe filter equipped with a 0.22-μm Millipore membrane filter. The CFS was subsequently stored at −80°C until further use.

### 2.3. In Vitro Antioxidant Activity Determination

#### 2.3.1. 2,2-Diphenyl-1-picrylhydrazyl (DPPH) Free Radical Scavenging Assay

The DPPH free radical scavenging activity of the bifidobacteria CFS was evaluated using a DPPH free radical scavenging assay kit (Yuanye, Shanghai, China) following the manufacturer's instructions. Briefly, the blank tube contained 0.05 mL of nitrogen radical extract and 0.45 mL of DPPH solution. The sample test tube contained 0.05 mL of bifidobacteria CFS and 0.45 mL of DPPH solution, while the sample control tube contained 0.05 mL of bifidobacteria CFS and 0.45 mL of absolute ethanol. The three tubes were mixed and incubated in the dark at room temperature for 30 min. Subsequently, 300 μL of the solution was used to measure the optical density at 517 nm (OD_517nm_) using a microplate reader (BioTek, Winooski, USA). The DPPH free radical scavenging rate was calculated based on the recorded measurements.

#### 2.3.2. Hydroxyl Free Radical Scavenging Assay

The hydroxyl free radical scavenging activity of the bifidobacteria CFS was evaluated using a hydroxyl free radical scavenging assay kit (Yuanye, Shanghai, China) following the manufacturer's instructions. Briefly, the blank tube contained 0.2 mL OH assay buffer and 0.8 mL distilled water. The undamaged tube contained 0.15 mL 1.10-phenanthroline solution, 0.2 mL OH assay buffer, 0.1 mL ferrous ion chromogenic solution, and 0.55 mL distilled water. The damaged tube contained 0.15 mL 1,10-phenanthroline solution, 0.2 mL OH assay buffer, 0.1 mL ferrous ion chromogenic solution, 0.45 mL distilled water, and 0.1 mL oxidizing agent. The sample control tube contained 0.2 mL OH assay buffer, 0.7 mL distilled water, and 0.1 mL bifidobacteria CFS. The sample test tube contained 0.15 mL 1,10-phenanthroline solution, 0.2 mL OH assay buffer, 0.1 mL ferrous ion chromogenic solution, 0.35 mL distilled water, 0.1 mL bifidobacteria CFS, and 0.1 mL oxidizing agent. The five tubes were incubated in a 37°C water bath for 60 min, and then, 250 μL of the solution was used to measure the OD_530nm_ using a microplate reader (BioTek, Winooski, USA). The hydroxyl radical scavenging rate was calculated based on the measurements obtained from the five tubes.

#### 2.3.3. Superoxide Anion Assay

The superoxide anion content was evaluated using a superoxide anion assay kit (Yuanye, Shanghai, China) following the manufacturer's instructions. Briefly, the blank tube contained 100 μL of distilled water. Six standard tubes contained 100 μL of NO_2_^−^ standard solution with concentrations of 10, 20, 30, 40, 50, or 60 μmol/L. The sample test tube contained 25 μL of bifidobacteria CFS, 25 μL of O_2_^−^ lysis buffer, and 50 μL of hydroxylamine solution. The eight tubes were mixed and incubated in a 25°C water bath for 20 min, after which 50 μL of aminobenzenesulfonic acid colorimetric solution and 50 μL of naphthylamine colorimetric solution were added to all tubes. The tubes were mixed and incubated in a 30°C water bath for 30 min. Subsequently, the OD_530nm_ was measured using a microplate reader (BioTek, Winooski, USA). The superoxide anion content was calculated based on the measurements obtained from the eight tubes.

#### 2.3.4. Total Antioxidant Capacity (2,2′-Azino-bis(3-ethylbenzothiazoline-6-sulfonic Acid [ABTS]) Assay

The total antioxidant capacity of the bifidobacteria CFS was evaluated using a total antioxidant capacity assay kit with the ABTS method (Beyotime, Shanghai, China), following the manufacturer's instructions. Briefly, the blank tube contained 200 μL of ABTS working solution and 10 μL of PBS. Six standard tubes contained 200 μL of ABTS working solution and 10 μL of Trolox standard solution with concentrations of 0.15, 0.3, 0.6, 0.9, 1.2, or 1.5 mmol/L. The sample test tube contained 200 μL of ABTS working solution and 10 μL of bifidobacteria CFS. The tubes were mixed and incubated at room temperature for 5 min. Subsequently, the OD_714nm_ was measured using a microplate reader (BioTek, Winooski, USA). The total antioxidant capacity was calculated based on the measurements obtained from the eight tubes.

### 2.4. In Vitro Antibacterial Activity Determination

#### 2.4.1. Agar Disc Diffusion Assay

The culture broth of *L. monocytogenes* was serially diluted to achieve a concentration of 10^6^ CFU/mL. A 100-μL aliquot of the solution was uniformly spread onto a Trypticase soy agar (TSA) plate (Huankai, Guangdong, China) using a coating rod, and then, a sterilized disc was adhered to the plate. Twenty microliters of bifidobacteria CFS was added to each corresponding disc (5 mm). An equal volume of fresh MRS broth (Solarbio, Beijing, China) served as the negative control. The zone of inhibition around each disc was measured after overnight incubation at 37°C.

#### 2.4.2. Antibacterial Growth Curve Assay

The CFS of each bifidobacteria strain was diluted to 100%, 80%, 60%, 40%, 20%, 10%, and 5% (v/v) with fresh MRS broth and transferred into a microplate with 200 μL per well. Subsequently, 20 μL of the previously diluted *L. monocytogenes* solution was added to each well. The growth curves were measured using the Bioscreen C automated system (Oy Growth Curves Ab Ltd, Helsinki, Finland) at 37°C. The OD_600nm_ of the bacterial suspensions was automatically recorded at 1-h intervals over a 27-h period. The cultures were automatically shaken for 60 s before each measurement. An equal volume of fresh MRS broth (Solarbio, Beijing, China) served as the negative control, while the diluted *L. monocytogenes* solution served as the positive control.

### 2.5. Coculture Assay

Activated *L. monocytogenes*, *B. breve*, *B. bifidum*, and *B. longum* subsp. *infantis* were adjusted to 10^9^ CFU/mL based on the corresponding absorbance and viable count curves before use. Three different inoculum ratios were studied. Briefly, *L. monocytogene*s was mixed with each bifidobacteria strain at ratios of 10:1, 1:1, or 1:10 (v/v) to prepare the mixtures. These mixtures were then co-incubated in an equal-volume mixed medium of MRS (Solarbio, Beijing, China) and TSB (Huankai, Guangdong, China) under anaerobic conditions at 37°C for 6 h as the test group. Individual cultures of *L. monocytogenes* under the same conditions were used as the control group. The viable counts of *L. monocytogenes* were determined using plate counts on chromogenic Listeria agar plates (Huankai, Guangdong, China).

### 2.6. Transcriptomics Analysis

Cocultures consisting of *L. monocytogenes* and *B. breve*, *B. bifidum*, and *B. longum* subsp. *infantis* at a ratio of 1:1 (v/v) were used as experimental groups, with individual cultures of *L. monocytogenes* serving as the control group for transcriptomics analysis. After incubation under the previously mentioned conditions, all groups were centrifuged at 12,000 rpm for 5 min, and the pellet was harvested and ground to a powder using liquid nitrogen. RNA was extracted using TRIzol reagent following the manufacturer's instructions (Thermo Fisher Scientific, Waltham, USA). RNA integrity and purity were assessed using a NanoDrop 2000 spectrophotometer (Thermo Fisher Scientific, Waltham, USA) and a Bioanalyzer 2100 (Agilent, Santa Clara, USA). RNA-seq transcriptome libraries were constructed using the TruSeqTM stranded total RNA library preparation kit following the manufacturer's instructions (Illumina, San Diego, CA). After rRNA was removed and mRNA was fragmented, cDNA was synthesized by reverse transcription. Library construction and RNA-seq data analysis were conducted by Majorbio (Shanghai, China) using the NovaSeq X Plus platform (Illumina, USA). Raw reads in FASTQ format were trimmed and quality-controlled using SeqPrep 2011 and Sickle 1.33. Clean, high-quality reads were aligned to the reference genome using Bowtie2 2.3.5. To identify differentially expressed genes (DEGs) between two samples, gene expression levels were calculated using RSEM 1.3.3 with the transcripts per million reads (TPM) method. Expression analysis considered a fold change (|log_2_FC| > 1) and a *p*-value < 0.05. The datasets pertinent to this investigation can be accessed in the National Center for Biotechnology Information (NCBI) BioProject repository with the accession identifier PRJNA1347567.

### 2.7. Real-Time qPCR

Selected DEGs were validated using real-time qPCR according to established protocols [16–18]. Briefly, total RNA was reverse transcribed using the PrimeScript FAST RT Reagent Kit with gDNA Eraser (Takara Bio, Beijing, China). qPCR amplification was performed on a Roche LightCycler 480 II system using FastStart Universal SYBR Green Master Mix (Rox, Roche Diagnostics). The thermal condition was set at 95°C for 10 min, followed by 45 cycles at 95°C for 25 s, 60°C for 60 s. Melt curve analysis was performed incrementally, raising the temperature from 72°C to 95°C with a ramp rate of 1°C per cycle. Gene expression levels were quantified using the 2^−ΔΔCt^ method with 16S *rRNA* serving as the endogenous control. The used primers are listed in Table 1.

### 2.8. Statistical Analyses

Statistical analyses and data presentation were performed using GraphPad Prism 8.0.1 software (GraphPad, Boston, USA). Data from independent experiments were expressed as mean ± SD, and statistical significance was determined using a one-sample *t*-test or one-way ANOVA followed by Tukey's honestly significant difference (HSD) procedure (*p* < 0.05). The experiments were conducted in triplicates.

## 3. Results

### 3.1. Comparative Analysis of In Vitro Antioxidant Activity

**Figure 1:**
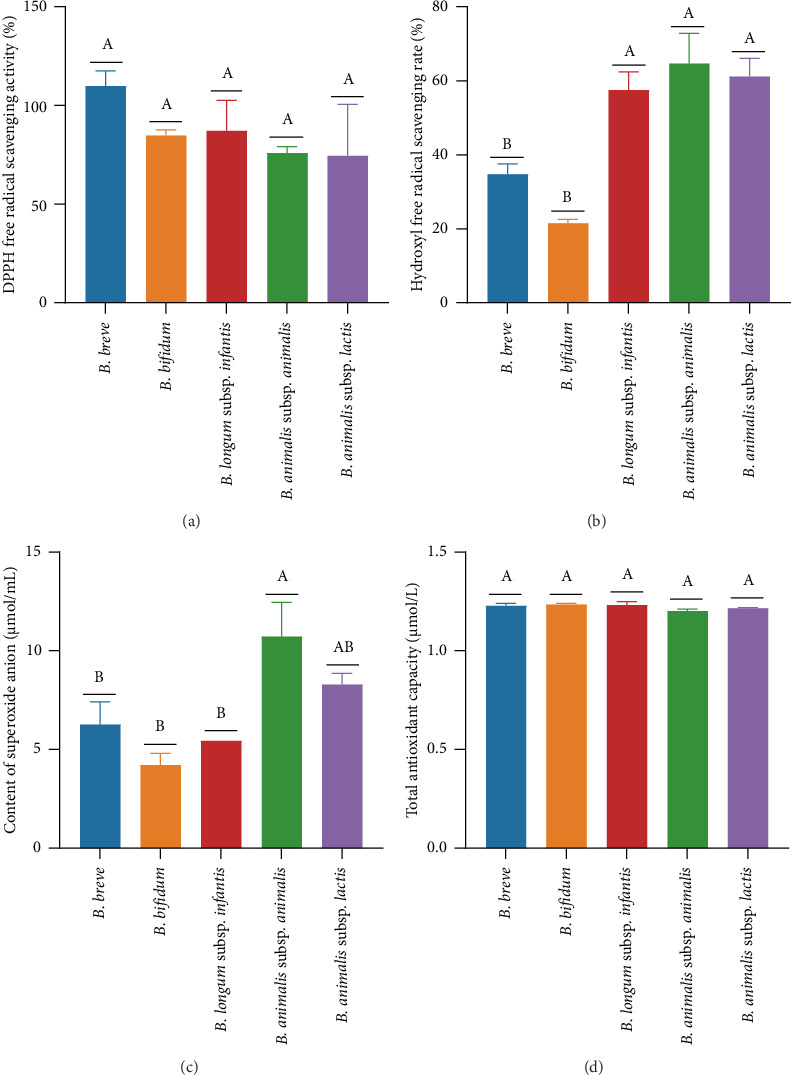
Comparative analysis of *in vitro* antioxidant activity. Note: (a) DPPH free radical scavenging rate; (b) hydroxyl free radical scavenging rate; (c) content of superoxide anion; (d) total antioxidant capacity. Different lowercase letters indicate significant differences between groups (*p* < 0.05), while the same letter indicates no significant difference (*p* > 0.05).

The *in vitro* antioxidant activities of five bifidobacteria CFSs, including DPPH free radical scavenging rate, hydroxyl free radical scavenging rate, superoxide anion content, and total antioxidant capacity, were compared. As shown in Figure 1(a), all bifidobacteria CFSs exhibited good DPPH free radical scavenging activities, with the following order: *B. breve* > *B. longum* subsp. *infantis* > *B. bifidum* > *B. animalis* subsp. *animalis* > *B. animalis* subsp. *lactis*. However, no significant difference was observed among the five strains (*p* > 0.05). As shown in Figure 1(b), the hydroxyl free radical scavenging rate of the bifidobacteria CFS followed the order: *B. animalis* subsp. *animalis* > *B. animalis* subsp. *lactis* > *B. longum* subsp. *infantis* > *B. breve* > *B. bifidum*, which differed significantly from the order observed for DPPH free radical scavenging activity. Interestingly, the *B. breve* CFS, with the highest DPPH free radical scavenging activity, exhibited significantly lower hydroxyl free radical scavenging activity compared to *B. animalis* subsp. *animalis*, *B. animalis* subsp. *lactis*, and *B. longum* subsp. *infantis* CFSs (*p* < 0.05). As shown in Figure 1(c), the superoxide anion content in the *B. animalis* subsp. *animalis* group was significantly higher than in the *B. breve*, *B. longum* subsp. *infantis*, and *B. bifidum* groups (*p* < 0.05), but no significant difference was observed with the *B. animalis* subsp. *lactis* group (*p* > 0.05). As shown in Figure 1(d), no significant differences were observed in the total antioxidant capacity among the five groups (*p* > 0.05). The results indicated that all five bifidobacteria strains exhibited definite *in vitro* antioxidant activities, but through different mechanisms.

### 3.2. Comparative Analysis of In Vitro Antibacterial Activity

**Figure 2:**
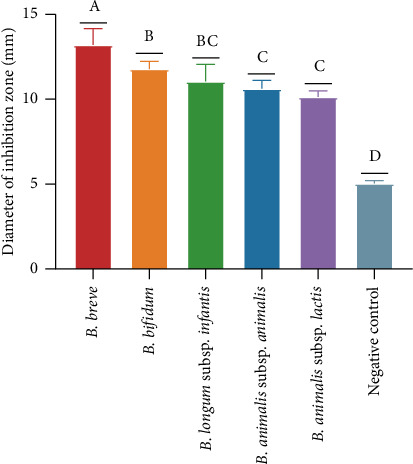
Comparative analysis of *in vitro* antibacterial activity using agar disc diffusion assay. Note: Different lowercase letters indicate significant differences between groups (*p* < 0.05), while the same letter indicates no significant difference (*p* > 0.05).

**Figure 3:**
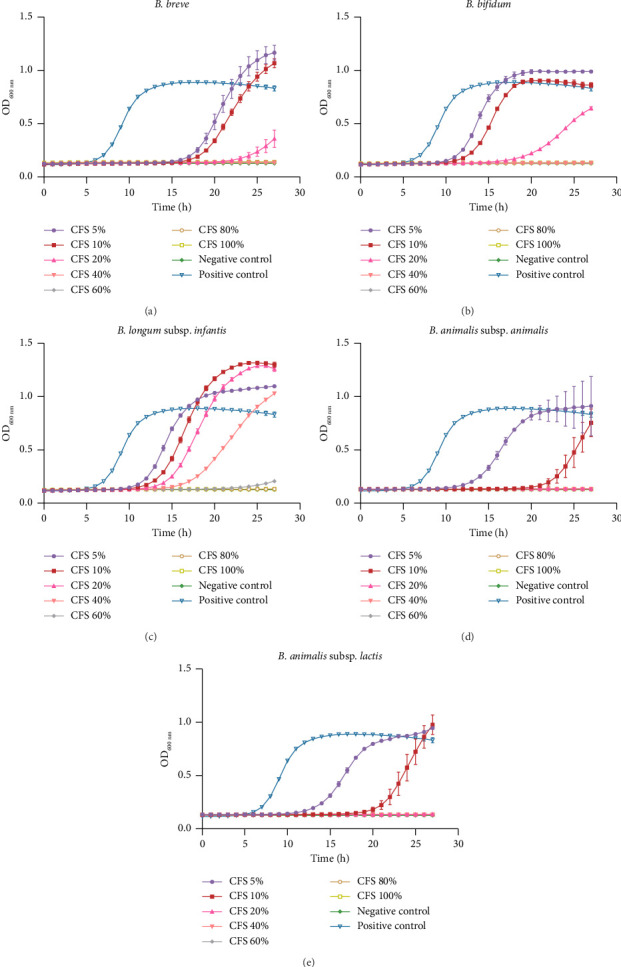
Comparative analysis of *in vitro* antibacterial activity using antibacterial growth curve assay. (a) *B. breve*; (b) *B. bifidum*; (c) *B. longum* subsp. *infantis*; (d) *B. animalis* subsp. *animalis*; (e) *B. animalis* subsp. *lactis*.

The *in vitro* antibacterial activity of five bifidobacteria CFSs was compared using both an agar disc diffusion assay (Figure 2) and an antibacterial growth curve assay (Figure 3). As shown in Figure 2, significant differences were observed in the diameter of the inhibition zone for all bifidobacteria CFSs against *L. monocytogenes* compared with the negative control group (*p* < 0.05), with the following order: *B. breve* > *B. bifidum* > *B. longum* subsp. *infantis* > *B. animalis* subsp. *animalis* > *B. animalis* subsp. *lactis*. As shown in Figure 3, all bifidobacteria CFSs exhibited clear inhibitory effects on *L. monocytogenes* in a dose-dependent manner, with the following order of inhibitory effects: *B. animalis* subsp. *animalis* > *B. animalis* subsp. *lactis* > *B. breve* > *B. bifidum* > *B. longum* subsp. *infantis*. Furthermore, even at a lower concentration (5%, v/v), the CFSs were able to delay the growth of *L. monocytogenes* until the logarithmic growth phase. Notably, *B. breve* delayed the entry of *L. monocytogenes* into the logarithmic growth phase to approximately 15 h, compared with 5 h for the positive control and 10 h for the other four bifidobacteria strains. These results demonstrated that all five bifidobacteria strains exhibited strong *in vitro* antibacterial activities against *L. monocytogenes*. Based on the comparison results, three representative strains, namely, *B. breve*, *B. bifidum*, and *B. longum* subsp. *infantis*, were selected for the coculture assay.

### 3.3. Effect of Coculture on *L. monocytogenes* Survival

The effects of coculture with different bifidobacteria strains on *L. monocytogenes* survival were investigated through viable counting (Table 2). Compared to individual culture, a slower growth rate of *L. monocytogenes* was observed in the presence of *B. breve*, *B. bifidum*, or *B. longum* subsp. *infantis*. Furthermore, their reduction rate was closely related to the ratio of coculture. The number of viable *L. monocytogenes* was significantly decreased (*p* < 0.05), especially when cocultured with any bifidobacteria strains at a ratio of 1:10 or even 1:1 (v/v). The results indicated that coculture with bifidobacteria strains may affect the survival of *L. monocytogenes*, suggesting that further attention to gene expression is needed.

### 3.4. Effect of Coculture on *L. monocytogenes* Gene Expression

#### 3.4.1. Patterns and Cluster Analysis of Gene Expression in Different Groups

**Figure 4:**
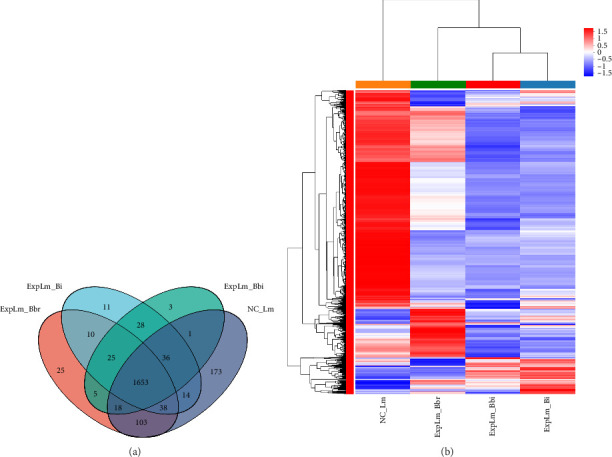
Patterns and cluster analysis of gene expression in different groups. (a) Venn; (b) Heatmap. Note: *ExpLm_Bbr*: experimental group cocultured with *L. monocytogenes* and *B. breve*; *ExpLm_Bbi*: experimental group cocultured with *L. monocytogenes* and *B. bifidum*; *ExpLm_Bi*: experimental group cocultured with *L. monocytogenes* and *B. longum* subsp. *infantis*; *NC_Lm*: negative control group with individual culture of *L. monocytogenes*.

RNA sequencing was employed to compare the gene expression patterns of *L. monocytogenes* cocultured with or without different bifidobacteria strains. The number of expressed genes in the control and three treated groups is shown in Figure 4(a). A total of 1653 genes were found to be commonly expressed in all four groups, while 25, 3, 11, and 173 genes were uniquely expressed in ExpLm_Bbr, ExpLm_Bbi, ExpLm_Bi, and NC_Lm groups, respectively. As shown in Figure 4(b), cluster analysis of these expression patterns was performed, which indicated that the expression levels of genes were significantly altered in all three experimental groups compared to the NC_Lm group. Furthermore, the trend of gene expression changes was more similar in the ExpLm_Bbi and ExpLm_Bi groups, followed by the ExpLm_Bbr group. In other words, fewer changes in *L. monocytogenes* gene expression levels were observed when cocultured with *B. breve* compared to *B. bifidum* and *B. longum* subsp. *infantis*. The results indicated that coculture with each bifidobacteria strain may cause significant changes in gene expression in *L. monocytogenes*, with the changes caused by *B. bifidum* and *B. longum* subsp. *infantis* being more prominent than those caused by *B. breve*.

#### 3.4.2. Differential Gene Expression Profile Analysis in Different Groups

**Figure 5:**
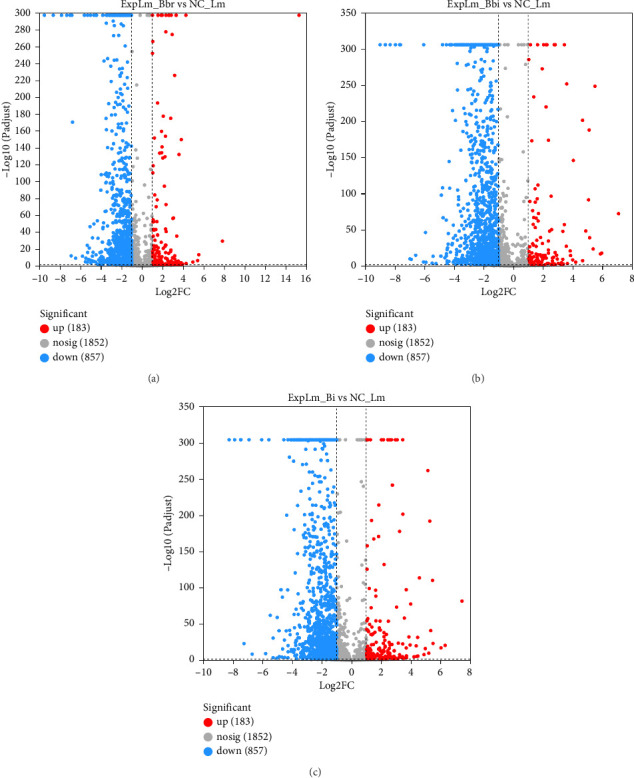
The number of DEGs of *L. monocytogenes* after coculture with different bifidobacteria strains. (a) ExpLm_Bbr vs NC_Lm; (b) ExpLm_Bbi vs NC_Lm; (c) ExpLm_Bi vs NC_Lm. Note: Blue points represent significantly downregulated DEGs, and red points represent significantly upregulated DEGs. *ExpLm_Bbr* vs *NC_Lm*: Gene sets of *L. monocytogenes* after coculture with *B. breve* compared to individual culture; *ExpLm_Bbi* vs *NC_Lm*: gene sets of *L. monocytogenes* after coculture with *B. bifidum* compared to individual culture; *ExpLm_Bi* vs *NC_Lm*: Gene sets of *L. monocytogenes* after coculture with *B. longum* subsp. *infantis* compared to individual culture.

**Figure 6:**
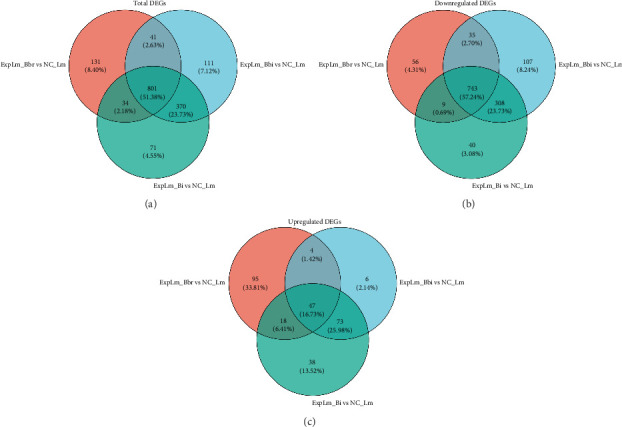
The number of unique and common of DEGs of *L. monocytogenes* across different gene sets. (a) Total DEGs; (b) downregulated DEGs; (c) upregulated DEGs.

As shown in Figure 5, after applying cutoffs for induction (ratio > 2.0-fold) and suppression (ratio < 0.5-fold), a total of 1040 DEGs, including 183 upregulated genes and 857 downregulated genes, were identified in the *B. breve* treatment group; a total of 1357 DEGs, including 134 upregulated genes and 1223 downregulated genes, were identified in the *B. bifidum* treatment group; and a total of 1317 DEGs, including 193 upregulated genes and 1124 downregulated genes, were identified in the *B. longum* subsp. *infantis* treatment group, respectively. Furthermore, 801 (51.38%) of the total number of DEGs were found to be common to all three treatment groups, and 131 (8.40%), 111 (7.12%), and 71 (4.55%) genes were uniquely expressed in the *B. breve*, *B. bifidum*, and *B. longum* subsp. *infantis* treatment groups, respectively (Figure 6(a)). Among them, 743 (52.24%) of the total number of downregulated DEGs were found to be common to all three treatment groups; 56 (4.31%), 107 (8.24%), and 40 (3.08%) genes were uniquely expressed in the *B. breve*, *B. bifidum*, and *B. longum* subsp. *infantis* treatment groups, respectively (Figure 6(b)); 47 (16.73%) of the total number of DEGs were found to be common to all three treatment groups; and 95 (33.81%), 6 (2.14%), and 38 (13.52%) genes were uniquely expressed in *B. breve*, *B. bifidum*, and *B. longum* subsp. *infantis*, respectively (Figure 6(c)). The results showed that consistent features of *L. monocytogenes* response to any bifidobacteria strains were mainly present, but specific features against *L. monocytogenes* also existed.

#### 3.4.3. Function Categories Related to Different Bifidobacteria Strain Interaction

**Figure 7:**
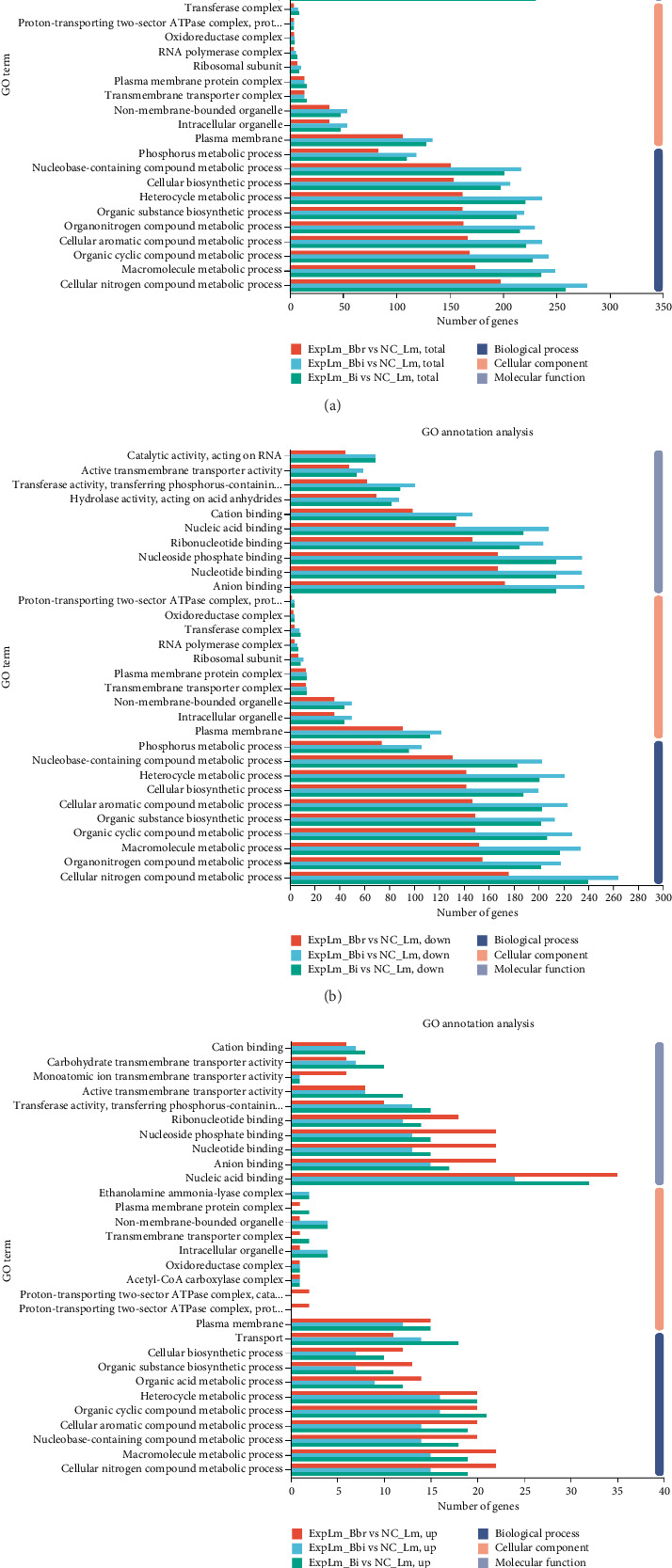
DEGs of *L. monocytogenes* affected by different bifidobacteria strains classified with GO database. (a) Total DEGs; (b) downregulated DEGs; (c) upregulated DEGs.

Based on the different transcriptional level analyses, DEGs were functionally characterized by comparison against the Gene Ontology (GO) database and classified into three categories: “biological process,” “cellular component,” and “molecular function,” as shown in Figure 7. The distribution trend of GO annotations in all three treatment groups appeared to be relatively consistent. As shown in Figure 7(a), the majority of DEGs were classified into “cellular nitrogen compound metabolic process GO:0034641,” “macromolecule metabolic process GO:0043170,” “organic cyclic compound metabolic process GO:1901360,” and others in the biological process category; “plasma membrane GO:0005886,” “intracellular organelle GO:0043229,” “non-membrane-bounded organelle GO:0043228,” and others in the cellular component category; and “anion binding GO:0043168,” “nucleoside phosphate binding GO:1901265,” “nucleotide binding GO:0000166,” and others in the molecular function category, which were similar to the downregulated DEGs shown in Figure 7(b). However, some unique categories of cellular components were also found in the upregulated DEGs, as shown in Figure 7(c). For the *B. breve* treatment group, several DEGs were categorized into “proton-transporting two-sector ATPase complex, catalytic domain GO:0033178” and “proton-transporting two-sector ATPase complex, proton-transporting domain GO:0033177,” but these categories were not found in the other two treatment groups. In contrast, the category “ethanolamine ammonia-lyase complex GO:0009350” was found in the *B. bifidum* and *B. longum* subsp. *infantis* treatment groups but not in the *B. breve* treatment group. Furthermore, two common categories, “plasma membrane protein complex GO:0098797” and “transmembrane transporter complex GO:1902495,” were found in the *B. breve* and *B. longum* subsp. *infantis* treatment groups but not in the *B. bifidum* treatment group. The results further suggested that different bifidobacteria strains may also have specific functional mechanisms against *L. monocytogenes* based on consistent features.

**Figure 8:**
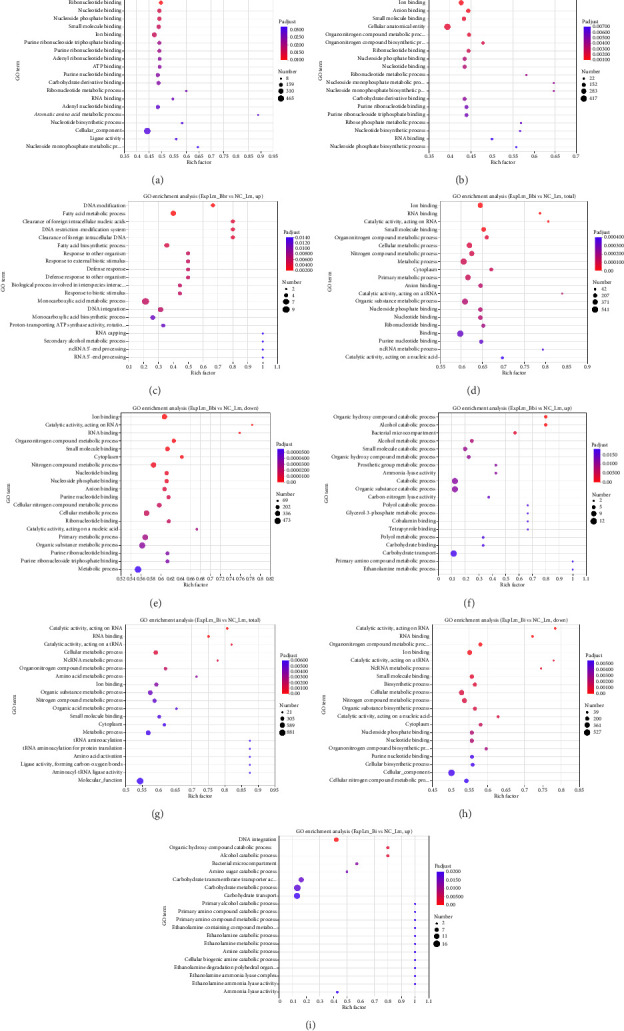
GO enrichment analysis of DEGs of *L. monocytogenes* affected by different bifidobacteria strains (a) Total DEGs, ExpLm_Bbr vs NC_Lm; (b) downregulated DEGs, ExpLm_Bbr vs NC_Lm; (c) upregulated DEGs, ExpLm_Bbr vs NC_Lm; (d) total DEGs, ExpLm_Bbi vs NC_Lm; (e) downregulated DEGs, ExpLm_Bbi vs NC_Lm; (f) upregulated DEGs, ExpLm_Bbi vs NC_Lm; (g) total DEGs, ExpLm_Bi vs NC_Lm; (h) downregulated DEGs, ExpLm_Bi vs NC_Lm; (i) upregulated DEGs, ExpLm_Bi vs NC_Lm.

GO enrichment analysis of DEGs was further performed, as shown in Figure 8. The top 20 enriched GO terms were displayed based on the *p*-value. As shown in Figure 8(a), for the *B. breve* treatment group, the majority of DEGs were enriched in various substance-binding categories, such as “anion binding GO:0043168,” “small molecule binding GO:0036094,” and “nucleotide binding GO:0000166,” which were similar to the downregulated DEGs shown in Figure 8(b), while most upregulated DEGs were enriched in “fatty acid metabolic process GO:0006631,” “clearance of foreign intracellular nucleic acids GO:0099046,” “response to other organisms GO:0051707,” and “defense response to other organisms GO:0098542” (Figure 8(c)). For the *B. bifidum* (Figures 8(d), 8(e)) and *B. longum* subsp. *infantis* (Figures 8(g), 8(h)) treatment groups, there were also quite a few downregulated DEGs enriched in various components binding, which indicated that these terms were common features of *L. monocytogenes* under bifidobacteria treatment. However, in these two treatment groups, many other downregulated (Figures 8(e), 8(h)) and upregulated (Figures 8(f), 8(i)) DEGs were enriched in multiple components of metabolic processes, which may suggest that *B. bifidum* and *B. longum* subsp. *infantis* were more capable of interfering with the metabolism of *L. monocytogenes* compared with *B. breve*.

#### 3.4.4. Pathways Related to Different Bifidobacteria Strain Interaction

**Figure 9:**
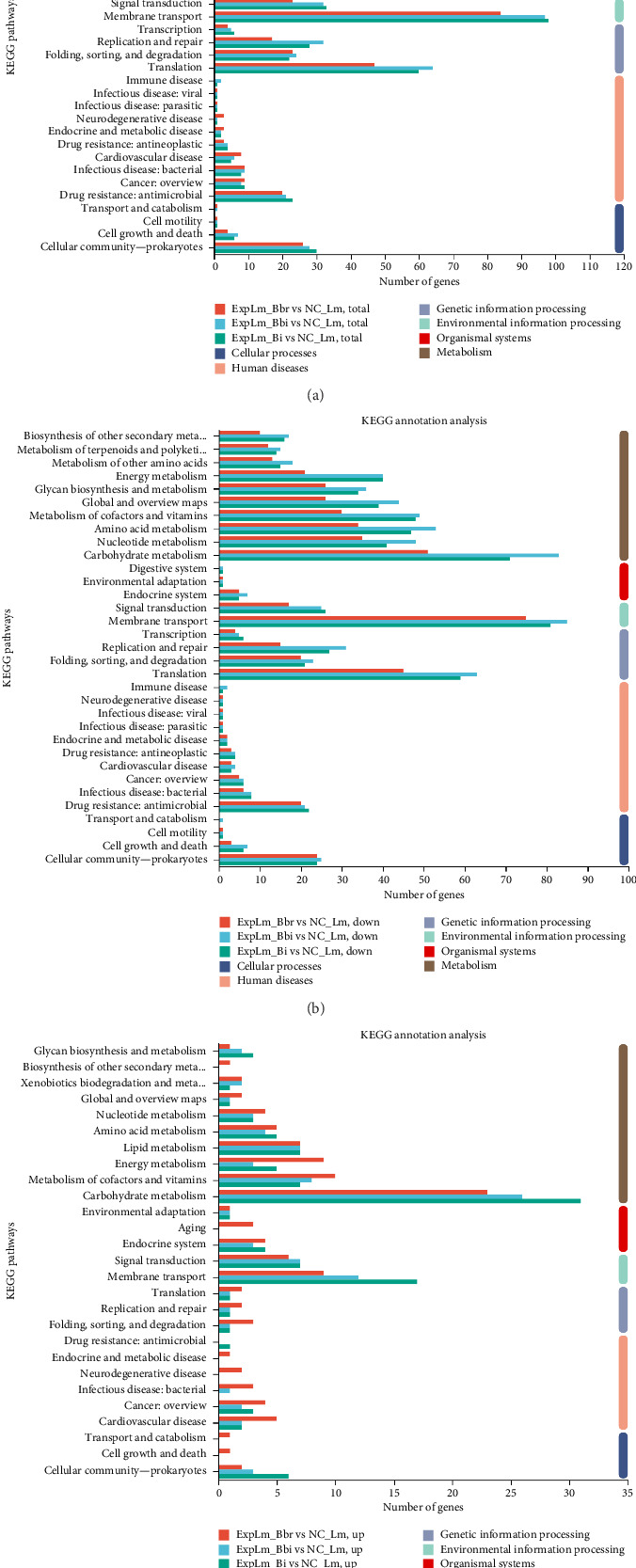
DEGs of *L. monocytogenes* affected by different bifidobacteria strains classified with KEGG database. (a) Total DEGs; (b) downregulated DEGs; (c) upregulated DEGs.

Based on the different transcriptional level analyses, DEGs were functionally characterized by comparison against the Kyoto Encyclopedia of Genes and Genomes (KEGG) database and classified into six pathway categories: “metabolism,” “organismal systems,” “environmental information processing,” “genetic information processing,” “human disease,” and “cellular processes,” as shown in Figure 9. The top 10 KEGG terms of each pathway category were displayed based on the number of genes. The distribution trend of KEGG annotations in all three treatment groups also appeared to be relatively consistent. As shown in Figure 9(a), the majority of DEGs were classified into pathways in the “metabolism” category, which is consistent with the GO annotations and enrichment results. Furthermore, several DEGs were enriched in other pathway categories, including “membrane transport” and “signal transduction” in the “environmental information processing” category, “translation” and “folding, sorting and degradation” in the “genetic information processing” category, “drug resistance: antimicrobial” and “infectious disease: bacterial” in the “human diseases” category, and “cellular community—prokaryotes” and “cell growth and death” in the “cellular processes” category. Similar trends were found in the downregulated DEGs, as shown in Figure 9(b). These common KEGG pathway categories may reflect consistent features of the inhibition mechanisms of *L. monocytogenes* by different bifidobacteria strains. Furthermore, some unique pathway categories were found in the upregulated DEGs, as shown in Figure 9(c). For example, several upregulated DEGs categorized into “biosynthesis of other secondary metabolites,” “cell growth and death,” and “transport and catabolism” were found only in the *B. breve* treatment group. Other upregulated DEGs categorized into “infectious disease: bacterial” were found in the *B. breve* and *B. bifidum* treatment groups but not in the *B. longum* subsp. *infantis* treatment group. In particular, another upregulated DEG categorized into “drug resistance: antimicrobial” was found only in the *B. longum* subsp. *infantis* treatment group. These unique KEGG pathway categories may reflect specific features of the inhibition mechanisms of *L. monocytogenes* by different bifidobacteria strains.

**Figure 10:**
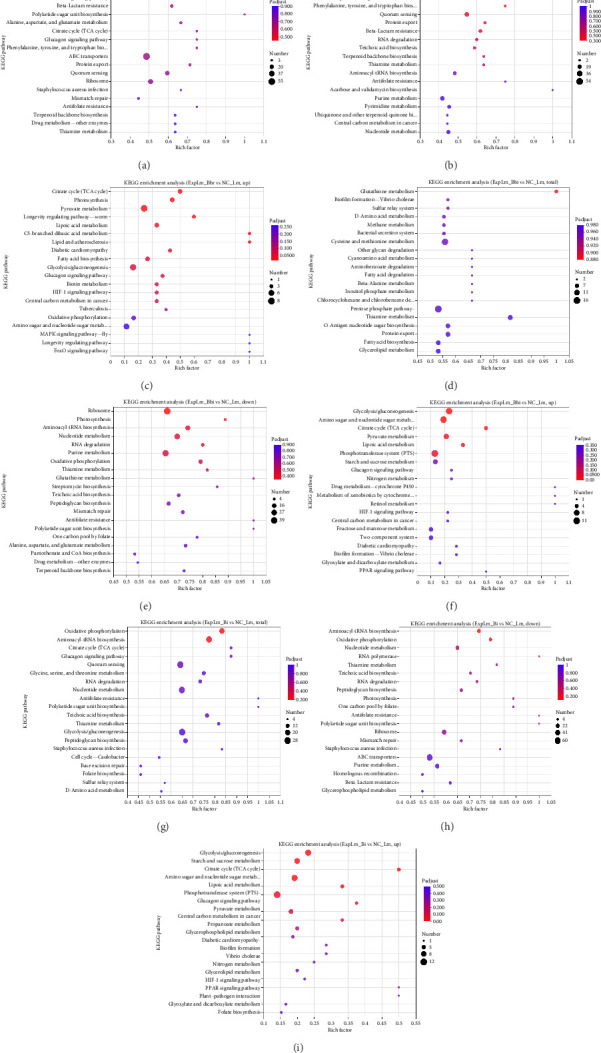
KEGG enrichment analysis of DEGs of *L. monocytogenes* affected by different bifidobacteria strains. (a) Total DEGs, ExpLm_Bbr vs NC_Lm; (b) downregulated DEGs, ExpLm_Bbr vs NC_Lm; (c) upregulated DEGs, ExpLm_Bbr vs NC_Lm; (d) total DEGs, ExpLm_Bbi vs NC_Lm; (e) downregulated DEGs, ExpLm_Bbi vs NC_Lm; (f) upregulated DEGs, ExpLm_Bbi vs NC_Lm; (g) total DEGs, ExpLm_Bi vs NC_Lm; (h) downregulated DEGs, ExpLm_Bi vs NC_Lm; (i) upregulated DEGs, ExpLm_Bi vs NC_Lm.

KEGG enrichment analysis of DEGs was further performed, as shown in Figure 10. The top 20 enriched KEGG terms were displayed based on the *p*-value. As shown in Figure 10(a), for the *B. breve* treatment group, the majority of DEGs were enriched in “map02010 ABC transporters,” “map03010 Ribosome,” and “map02024 Quorum sensing,” which were similar to the downregulated DEGs shown in Figure 10(b), while most upregulated DEGs were enriched in various metabolic processes, including “map00620 Pyruvate metabolism,” “map00010 Glycolysis/Gluconeogenesis,” “map00020 Citrate cycle (TCA cycle),” and “map00785 Lipoic acid metabolism” (Figure 10(c)). For the B. bifidum treatment group, as shown in Figure 10(d), the majority of DEGs were enriched in “map00480 Glutathione metabolism,” “map05111 Biofilm formation—*Vibrio cholerae*,” “map03070 Bacterial secretion system,” and “map00270 Cysteine and methionine metabolism.” The downregulated DEGs were enriched in biosynthesis and metabolism pathways, such as “map03010 Ribosome,” “map00195 Photosynthesis,” and “map00550 Peptidoglycan biosynthesis” (Figure 10(e)), and most upregulated DEGs were enriched in “map02060 Phosphotransferase system (PTS),” “map00010 Glycolysis/Gluconeogenesis,” “map00520 Amino sugar and nucleotide sugar metabolism,” and “map00020 Citrate cycle (TCA cycle)” (Figure 10(f)). For the *B. longum* subsp. *infantis* treatment group, as shown in Figure 10(g), several DEGs were enriched in “map00010 Glycolysis/Gluconeogenesis,” “map02024 Quorum sensing,” and “map00190 Oxidative phosphorylation.” Its downregulated DEGs were mainly enriched in “map02010 ABC transporters” and “map03010 Ribosome,” which is consistent with the *B. breve* treatment group (Figure 10(h)), while the upregulated DEGs were enriched in “map02060 PTS,” “map00010 Glycolysis/Gluconeogenesis,” and “map00520 Amino sugar and nucleotide sugar metabolism,” which is consistent with the *B. bifidum* treatment group (Figure 10(i)). The results further indicated that a general antibacterial mechanism exists in different bifidobacteria strains, but also with a few of their own characteristics.

#### 3.4.5. Virulent Factor (VF) Gene Expression Related to Different Bifidobacteria Strain Interaction

**Figure 11:**
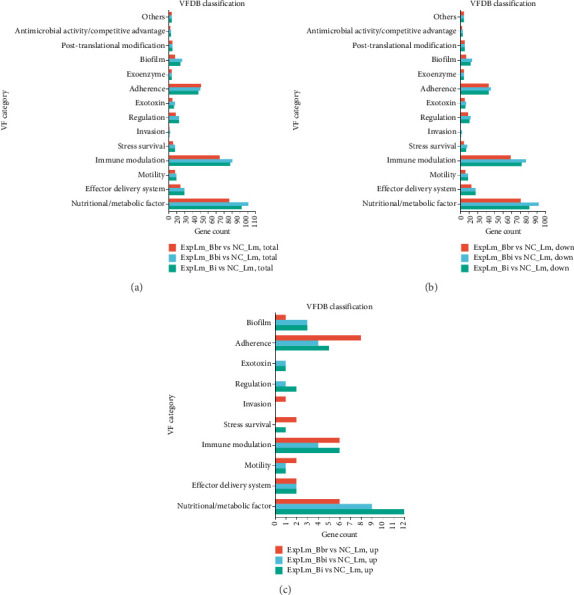
Virulent factor DEGs of *L. monocytogenes* affected by different bifidobacteria strains classified with VFDB database. (a) Total DEGs; (b) downregulated DEGs; (c) upregulated DEGs.

DEGs were classified into fourteen different VF categories, namely, “Nutritional/Metabolic factor,” “Effector delivery system,” “Motility,” “Immune modulation,” “Stress survival,” “Invasion,” “Regulation,” “Exotoxin,” “Adherence,” “Exoenzyme,” “Biofilm,” “Post-translational modification,” “Antimicrobial activity/Competitive advantage,” and “Others” by comparison against the VFDB database based on different transcriptional level analyses, as shown in Figure 11. As shown in Figure 11(a), the distribution trend of VF annotations for total DEGs in all three treatment groups appeared to be relatively consistent, with the number of DEGs being notably lower in the *B. breve* than *B. bifidum* and *B. longum* subsp. *infantis* treatment group. Similar trends were found in downregulated DEGs as shown in Figure 11(b), while the upregulated DEGs classified into several VF categories including “Stress survival,” “Invasion,” “Regulation,” and “Exotoxin” were evidently different, as shown in Figure 11(c). The results indicated that coculture with any of the three bifidobacteria strains could affect the transcriptomic response of *L. monocytogenes* key virulence genes with common but also specific characteristics.

**Figure 12:**
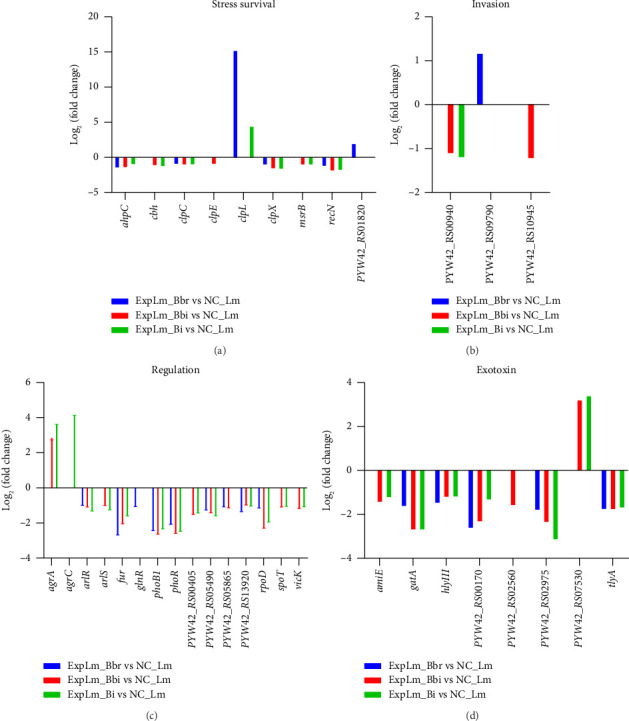
Fold changes of DEGs of *L. monocytogenes* classified into several special VF categories (a) stress survival; (b) invasion; (c) regulation; (d) exotoxin.

Furthermore, the fold changes of DEGs of *L. monocytogenes* classified into these special VF categories are shown in Figure 12. As shown in Figure 12(a), the transcriptional levels of *ahpC*, *clpC*, *clpX*, and *recN* were downregulated in all three treated groups, indicating that the three bifidobacteria strains could inhibit the survival of *L. monocytogenes* through the suppression of these stress genes. However, the expression of *cbh* and *msrB* was significantly downregulated in the *B. bifidum* and *B. longum* subsp. *infantis* treatment group, but not in the *B. breve* treatment group, and the downregulated expression of *clpE* was found only in the *B. bifidum* treatment group.

**Figure 13:**
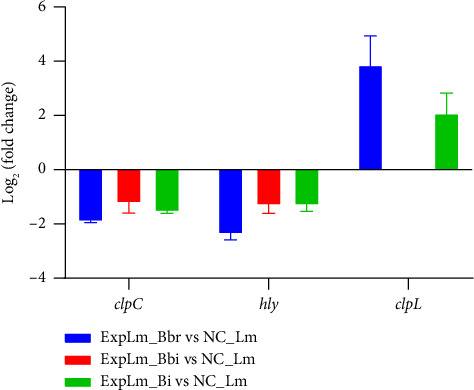
Validation of the representative DEGs detected in the transcriptomic analysis using real-time qPCR.

Interesting, *clpL* was found to be upregulated in both the *B. breve* and *B. longum* subsp. *infantis* treatment group, and *PYW42_RS01820* was upregulated in the *B. breve* treatment group. As shown in Figure 12(b), *PYW42_RS00940*, *PYW42_RS09790*, and *PYW42_RS10945* were all required for the entry of *L. monocytogenes* into nonphagocytic cells and were necessary for full virulence. For the *B. bifidum* treatment group, the expression of *PYW42_RS00940* and *PYW42_RS10945* was significantly suppressed. For the *B. longum* subsp. *infantis* treatment group, the expression of *PYW42_RS00940* was also suppressed, while the expression of *PYW42_RS09790* was enhanced in the *B. breve* treatment group. As shown in Figure 12(c), the majority of DEGs including *arlR*, *fur*, *phoB1*, *phoR*, *PYW42_RS05490*, *PYW42_RS13920*, and *rpoD* were similarly expressed in the three treatment groups, with a noticeably smaller number of DEGs classified into “Regulation” VF category that was less in the *B. breve* treatment group. As shown in Figure 12(d), the expression of “Exotoxin” DEGs was most suppressed, such as *amiE*, *gatA*, *PYW42_RS02975*, and *tlyA*, *hlyIII*, which indicated that bifidobacteria strains could inhibit the expression of *L. monocytogenes* key virulence genes. Furthermore, three representative DEGs including *clpC*, *hly*, and *clpL* were validated by real-time qPCR. As shown in Figure 13, the real-time qPCR results demonstrated expression patterns consistent with the RNA-seq findings, confirming the reliability of our transcriptomic analysis.

## 4. Discussion

The genus *Bifidobacterium* is well known as a safe and effective probiotic for its health-promoting effects [19]. The colonization of bifidobacteria strains is believed to play pivotal roles in the maturation of the immune, digestive, and metabolic systems, thereby protecting against susceptibility to various diseases in humans across their lifespan [20]. Various studies have suggested that bifidobacteria strains and their metabolites can maintain and promote intestinal health [21–23]. For example, several studies have demonstrated that bifidobacteria antioxidant components can protect against diseases associated with oxidative stress, such as inflammatory bowel diseases [22]. Yun et al. [23] found that *B. longum* could repress infectious diseases caused by *Clostridium difficile*. Therefore, in the present study, we first investigated the *in vitro* antioxidant and antibacterial activities of five bifidobacteria CFSs. The results demonstrated that all five bifidobacteria strains exhibited excellent *in vitro* antioxidant capacity and antibacterial activity against *L. monocytogenes* (Figures 1 and 2), consistent with the studies by Ma [24] and Muñoz-Quezada et al. [25]. Furthermore, *Bifidobacterium* species can be categorized into infant-type human-residential bifidobacteria (HRB), adult-type HRB, and non-HRB based on their residential origins [26]. *B. breve*, *B. bifidum*, and *B. longum* subsp. *infantis* are referred to as infant-type HRB, as they are the dominant species in an infant's intestines [27]. *B. animalis* subsp. *animalis* and *B. animalis* subsp. *lactis* are referred to as non-HRB, as they are the natural inhabitants of animals or the environment [28]. The species of HRB and non-HRB have been recognized to display differences in their ecological adaptation and physiological properties relevant to human health [29]. In the present study, three types of infant-type HRB along with two types of non-HRB were selected to compare their probiotic characteristics. The results indicated that the three infant-type HRB strains showed a better DPPH free radical scavenging rate (Figure 1(a)) and larger inhibition zones against *L. monocytogenes* (Figure 2) than the two non-HRB strains. Ma et al. [24] showed that the CFS of *B. longum* subsp. *infantis* strain YLGB-1496 exhibited strong free radical scavenging activity against DPPH, ABTS, ·OH, and O_2-_ radicals. Research has indicated that *B. breve* CNCM I-4035 can inhibit the *in vitro* survival and growth of *L. monocytogenes* [25]. Lin [30] found that *B. bifidum* BGN4-SK has enhanced antioxidant and anti-inflammatory activities. These findings indicate that these infant-type HRB strains have greater potential for preventing and treating *L. monocytogenes* infections and promoting maternal and fetal health. Therefore, we further investigated their effects on the key gene expression of *L. monocytogenes* using a coculture assay.

Coculture studies are valuable for understanding the interactions between microorganisms. Cecil et al. [31] examined the influence of environmental factors on *Acanthamoeba castellanii* and *Pseudomonas aeruginosa* in coculture to better explore interspecies interactions. Bradford et al. [32] effectively screened probiotic strains that inhibit adherent-invasive *Escherichia coli* using a coculture system. Qiao et al. [33] investigated the motility, biofilm formation, and associated gene expression in *Vibrio parahaemolyticus* impaired by coculture with live *Ulva fasciata*. Previous studies have investigated the effects on the key gene expression of *L. monocytogenes* during coculture with various microorganisms [16, 34]. Lappa et al. [34] found that the *L. monocytogenes sigB* gene was downregulated after coculture with *Aspergillus flavus* by RT-qPCR. However, the global transcriptomic profiles of *L. monocytogenes* under coexistence with infant-type HRB strains are currently unclear. Recently, transcriptomic profiling techniques have been used to enhance our understanding of the mechanisms underlying microorganisms' and hosts' interactions [35]. Cortes et al. [36] identified the *σB* regulon as potentially involved in the response to acid exposure by examining the transcriptomes of *L. monocytogenes* under lactic acid stress. Liao et al. [37] investigated the inhibition mechanism of protocatechualdehyde against *L. monocytogenes* using transcriptome analysis. In the present study, a comparative transcriptomic analysis of *L. monocytogenes* in response to *B. breve*, *B. bifidum*, and *B. longum* subsp. *infantis* interactions was performed using RNA-seq. The results indicated that the three infant-type HRB strains could inhibit cell growth (Table 2) and alter various gene expressions (Figure 4) of *L. monocytogenes*, consistent with our previous study [16].

Through quantitative results of RNA-seq, DEGs of *L. monocytogenes* exposed to different infant-type HRB strains were identified and compared (Figure 5). The results indicated that more than half of the DEGs (both up- and downregulated) were common to all three treatment groups, suggesting that different bifidobacteria strains exhibit a similar mechanism against *L. monocytogenes* (Figure 6). For instance, according to the GO results, “anion binding GO:0043168,” “small molecule binding GO:0036094,” and “response to other organism GO:0051707” were enriched in common DEGs (Figures 7 and 8), which may represent the identified targets related to anti–*L. monocytogenes* biological processes [38, 39]. According to the KEGG results, such as “map02010 ABC transporters,” “map02024 Quorum sensing,” “map00550 Peptidoglycan biosynthesis,” and “map00480 Glutathione metabolism” were enriched in common DEGs (Figures 9 and 10), which may indicate potential common mechanisms by which infant-type HRB strains control *L. monocytogenes* [40–43]. ABC transporters have been demonstrated to be involved in virulence and stress responses, and are considered antibacterial targets [40]. Quorum sensing is a cell-to-cell communication process that controls biofilm formation and virulence, with many antibacterial agents inhibiting *L. monocytogenes* by interfering with quorum-sensing systems [41]. The normal synthesis of peptidoglycan is essential for maintaining bacterial morphology, and numerous antibiotics target peptidoglycan synthases [42]. Glutathione metabolism regulates the PrfA expression of *L. monocytogenes*, and disruption of this process impairs virulence program expression [43].

Furthermore, the effects of the three infant-type HRB strains on key VF gene expression of *L. monocytogenes* were in particular surveyed and compared (Figures 11 and 12). The results indicated that *ahpC*, *clpC*, *clpX*, *recN*, *arlR*, *rpoD*, *tlyA*, and *hlyIII* were the common hub genes of *L. monocytogenes* in response to infant-type HRB interactions. The *ahpC* gene is a common antioxidant gene of *L. monocytogenes* that can be inactivated by cold plasma, which is consistent with the findings of our study [44]. The *clpC* gene encodes an Hsp100/Clp protein, which forms an ATP-dependent protease by associating with the peptidase ClpP and contributes to the virulence and stress tolerance of *L. monocytogenes* [45]. The *clpX* gene encodes the AAA + chaperone ClpX, which binds with ClpP to enhance chaperone affinity and substrate turnover in *L. monocytogenes* [46]. RecN is a cohesin-like protein in bacteria that plays a crucial role in maintaining genomic integrity by facilitating the repair of DNA double-strand breaks. The cell survival of *recN*-deletion bacterial mutants is reduced when exposed to DNA-damaging agents [47]. ArlR consists of an N-terminal receiver domain and a C-terminal DNA-binding effector domain, which regulates bacterial adhesion, biofilm formation, and virulence [48]. The rpoD gene encodes RNA polymerase sigma 70, which is significantly upregulated during infection and could serve as a potential target for inhibition of *L. monocytogenes* [49]. TlyA is characterized as a putative pore-forming cytolysin in multiple pathogens, including *Helicobacter pylori*, *Brachyspira hampsonii*, and *Mycobacterium* spp. [50–52]. The *hlyIII* gene is also a vital virulence gene involved in hemolysis and cytotoxicity [53]. These findings may indicate that infant-type HRB strains with strong antioxidant and antibacterial activities could interfere with synthesis and metabolism, thereby enhancing the susceptibility of *L. monocytogenes* as their common features. In addition, the specific DEGs of *L. monocytogenes* in different infant-type HRB-treated groups reflect strain-specific virulence and stress sensitivity mechanisms. For instance, MsrB is responsible for reducing oxidized methionine, and its mutant shows decreased *in vitro* growth, resistance to exogenous oxidative stress, and reduced intracellular growth in macrophages [54]. However, in our study, we found that the expression of *msrB* was significantly suppressed when exposed to *B. bifidum* and *B. longum* subsp. *infantis*, but not *B. breve*. The expression trends of *amiE*, a novel N-acylhomoserine lactone acylase belonging to the amidase family and acting as a quorum-sensing signal molecule, were similar to *msrB* [55]. The specific DEG *clpE* was affected only by *B. bifidum*, which acts synergistically with ClpC and plays a crucial role in both cell division and virulence of *L. monocytogenes* [56]. In contrast, the expression of *clpL*, a potent stand-alone disaggregase enabling *L. monocytogenes* persistence in adverse environments, was upregulated in both *B. breve* and *B. longum* subsp. *infantis* treatment groups, but not in the *B. bifidum* treatment group [57].

Nevertheless, *Listeria monocytogenes* exhibits the capacity to form biofilms on various surfaces, conferring enhanced resistance to disinfectants and posing significant public health concerns [58]. Current research reveals that lactic acid bacteria exhibit potent antibiofilm activity against *L. monocytogenes* through multiple inhibitory mechanisms [59]. Particularly noteworthy is the involvement of quorum-sensing systems, which coordinately regulate both biofilm development and virulence expression in this pathogen [60]. Our KEGG pathway analysis identified significant enrichment of “map02024 Quorum sensing” among common DEGs (Figures 9 and 10), strongly supporting the hypothesis that the three infant-type HRB strains attenuate *L. monocytogenes* motility and biofilm formation via QS interference. Future investigations need to focus on comprehensive characterization of strain-specific antimicrobial metabolites, and systematic evaluation of their antibiofilm efficacy against *L. monocytogenes*.

## 5. Conclusions

The findings of the current study suggest that the bifidobacteria strains used exhibit distinct *in vitro* antioxidant and antibacterial activities against *L. monocytogenes*, particularly the infant-type HRB strains. A comprehensive analysis of transcript-level differences in *L. monocytogenes* in response to the three infant-type HRB strains was conducted. Our results indicate that co-incubation with these infant-type HRB strains resulted in decreased survival of *L. monocytogenes* and significant changes in the expression levels of key genes. By carefully categorizing DEGs, we examined the overall and specific gene networks among the three infant-type HRB strains to uncover both their similarities and differences in mechanisms of antagonism. Our analysis revealed that a large proportion of DEGs were common across these three treatment groups, suggesting potential shared mechanisms through which infant-type HRB strains regulate *L. monocytogenes*. These hub DEGs were enriched in the following GO terms: “anion binding GO:0043168,” “small molecule binding GO:0036094,” and “response to other organism GO:0051707,” and KEGG terms: “map02010 ABC transporters,” “map02024 Quorum sensing,” “map00550 Peptidoglycan biosynthesis,” and “map00480 Glutathione metabolism,” Moreover, key virulence-related DEGs targeting *L. monocytogenes* were specifically examined and compared. Our investigation identified *ahpC*, *clpC*, *clpX*, *recN*, *arlR*, *rpoD*, *tlyA*, and *hlyIII* as common hub DEGs in response to interaction with infant-type HRB strains. Additionally, several unique DEGs, such as *msrB*, *amiE*, *clpE*, and *clpL*, reflecting strain-specific virulence and stress sensitivity mechanisms, were identified among the different treatment groups. These findings not only enhance our understanding of probiotics–pathogen interactions but also contribute to the development of targeted antagonistic approaches for controlling *L. monocytogenes*.

## Figures and Tables

**Table 1:** Primers used in real-time qPCR.

Target gene	Sequence (5′–3′)	Source
*clpC*	F: GCGGCTGTTCAAGGKCAAG R: TTGCCAATTCGCTTTWGTTTCTT	[17]
*hly*	F: CATAGCACCACCAGCATCTCC R: TGTCACTGCATCTCCGTGG	[16]
*clpL*	F: TGGGCACTTAACGGATGGAC R: TGATCTCGTTGCTCGTCACC	[18]
*16S rRNA*	F: TTTAGTTGCCAGCATTTAGTTGG R: GTGTGTAGCCCAGGTCATAAGG	[16]

**Table 2:** Impacts of different bifidobacteria strains on *L. monocytogenes* survival during their coculture for 6 h.

Group	Ratio	Viable counts of *L. monocytogenes*
Control	Individual culture	8.61 ± 0.05^a^

Coculture of *L. monocytogenes* and *B. breve*	10:1	8.55 ± 0.12^∗a^
	1:1	6.24 ± 0.08^#b^
	1:10	5.90 ± 0.18^c^

Coculture of *L. monocytogenes* and *B. bifidum*	10:1	7.98 ± 0.01^∗b^
	1:1	6.88 ± 0.05^#c^
	1:10	5.92 ± 0.04^d^

Coculture of *L. monocytogenes* and *B. longum* subsp. *infantis*	10:1	8.29 ± 0.08^∗b^
	1:1	6.45 ± 0.06^#c^
	1:10	5.59 ± 0.04^†d^

*Note:* Different lowercase letters represent significant differences between different groups (*p* < 0.05); the same letter represents no significant difference between different groups (*p* > 0.05).

^∗^a significant difference in the viable counts of *L. monocytogenes* when comparing the groups of three bifidobacteria strains at a 10:1 ratio (*p* < 0.05).

^#^a significant difference in the viable counts of *L. monocytogenes* when comparing the groups of three bifidobacteria strains at a 1:1 ratio (*p* < 0.05).

^†^a significant difference in *L. monocytogenes* counts between the *B. longum* subsp. *infantis* group and the other two bifidobacteria strains (*B. breve* and *B. bifidum*) at a 1:10 ratio.

## Data Availability

The data that support the findings of this study are available from the corresponding author upon reasonable request.
